# Metals Transfer in Mushroom *Tricholoma matsutake* from Regional High Geochemical Background Areas: Environmental Influences and Human Health Risk

**DOI:** 10.3390/jof10090608

**Published:** 2024-08-26

**Authors:** Cuiting Wang, Jue Bi, Yukang Zhang, Yixuan Zhang, Xue Liu

**Affiliations:** 1 Yunnan Key Laboratory of Plateau Wetland Conservation, Restoration and Ecological Services, Southwest Forestry University, Kunming 650224, China; wct20220913@126.com (C.W.); zhangyukang818@126.com (Y.Z.); zhangyixuan202303@163.com (Y.Z.); 2 Institute of Tropical and Subtropical Cash Crops, Yunnan Academy of Agricultural Sciences, Baoshan 678000, China; jue_bi1995@126.com

**Keywords:** *Tricholoma matsutake*, soil, metal, bioaccumulation, health risk assessment

## Abstract

Wild-grown edible mushrooms are important in world diets and are also efficient metal accumulators. Yunnan, Southwest China, is the main producing region, with typically high levels of geochemical metals. The environmental factors, bioaccumulation, distribution and human health risks of metals were examined in paired soil and *Tricholoma matsutake* (n = 54). *T*. *matsutake* grows on acidified soils (pH = 3.95–6.56), and metals show a strong heterogeneity, with Fe, Mn, Zn and Cu in the ranges of 16–201, 0.046–8.58 g kg^−1^, and 22.6–215, 3.7–155 mg kg^−1^. High soil Fe content led to great accumulation in *T*. *matsutake* (0.24–18.8 g kg^−^^1^). However, though the soil Mn content was higher than that of Zn and Cu, their concentrations in *T*. *matsutake* were comparable (21.1–487 vs. 38.7–329 and 24.9–217 mg kg^−^^1^). This suggested that *T*. *matsutake* prefers to accumulate Zn and Cu compared to Mn, and this is supported by the bioaccumulation factors (BAFs = 0.32–17.1 vs. 0.006–1.69). Fe was mainly stored in stipes, while Mn, Zn and Cu were stored in caps, and the translocation factors (TFs) were 0.58 vs. 1.28–1.94. Therefore, stipe Fe showed the highest health risk index (HRI) at 1.28–26.9, followed by cap Cu (1.01–2.33), while 98–100% of the Mn and Zn were risk-free. The higher concentration and greater risk of Fe was attributed to the significant effect of soil Fe content (R = 0.34) and soil pH (R = −0.57). This study suggested that Fe, as an essential mineral, may exert toxic effects via the consumption of *T*. *matsutake* from high geochemical background areas.

## 1. Introduction

Generally, mushrooms are potential sources of bioactive compounds like vitamin B2, minerals and antioxidants (ergothioneine and glutathione), which have pro-health properties, including anticancer, antidiabetic and immunomodulating activities. Typically, wild edible mushrooms play an important role in world diets, with consumption steadily increasing due to their texture, flavor and nutritional and medicinal value, especially in Europe and Asia [1,2]. Specifically, 73.5% of mushroom consumers in the Czech Republic have a local consumption of up to 1.0 kg per capita and 5.6 kg per household annually [3,4]. Yunnan Province, located in Southwest China, is rich in wild edible mushrooms and is the major distribution and production region [5]. It accounts for 91% of the known abundance of wild edible mushrooms in China and 43% of the world abundance [6]. *Tricholoma matsutake* is the most valuable, frequently consumed and economically important species [7,8], whose terpenoid and polysaccharide extracts show antitumor and antioxidant values [9,10]. Additionally, the export rate of *T*. *matsutake* from Yunnan accounts for 80% of the total exports in China [11].

In addition to the abundance of mushrooms, Yunnan is characterized by diverse polymetallic bedrocks and high geochemical background concentrations of metals. Furthermore, Yunnan has abundant mineral resources and is known as “the kingdom of nonferrous metals”. The high concentrations of background metals together with the intensive mining activities lead to soils being heavily polluted and thereby to potential transfer via the food chain, thus causing risk to humans. Moreover, wild mushrooms are efficient metal accumulators, showing greater metal accumulations than common agricultural crop plants and vegetables [6]. Typically, *T*. *matsutake* can accumulate toxic metal(loid)s like cadmium (Cd), arsenic (As) and lead (Pb) in quantities of up to 2.88, 7.12 and 8.63 mg kg^−^^1^ [6,12]. Indeed, hazardous metals and nutritional components (proteins, vitamins and antioxidants) in *T*. *matsutake* have been studied in depth [11]. However, there are limited studies on the mineral metal accumulation and potential health risks of *T*. *matsutake* from areas with elevated geogenic metals.

Metals (Fe, Mn, Zn and Cu) are essential mineral components for human health [1], which, however, exert toxic effects when exceeding the amounts required for physiological functions [13]. Wild mushroom consumption is an important dietary source of mineral metals, which may pose a risk to human health. Generally, metal transfer and accumulation in mushrooms depend on the total concentration and soil physicochemical properties. Among soil properties, pH and organic matter (OM) are predominant in affecting metal bioavailability and thereby accumulation in mushrooms [14]. However, studies have mainly focused on the mineral concentration [15,16,17], composition and nutritional values, and limited information is available about the process of transfer, distribution, potential health risks and underlying influencing factors.

Therefore, the aims of this study were to (1) analyze the metals (Fe, Mn, Zn and Cu) concentrations and distributions (in caps and stipes) in paired soils and *T*. *matsutake* (n = 54) from Yunnan Province, China; (2) evaluate metal soil-to-fruiting body accumulation and stipe-to-cap transfer efficiency, and clarify the correlation between soil metal concentration, pH and OM with respect to *T*. *matsutake*; and (3) assess the edible safety by calculating the metal daily intake (DI) via *T*. *matsutake* ingestion and the associated health risk index (HRI). This study helps us to better understand the uptake, transfer, accumulation, potential health risks and influencing factors of mineral metals in wild mushrooms and provides hazard-level indications and requirements for exposure control awareness.

## 2. Materials and Methods

### 2.1. Sample Collection and Pretreatment

**Figure 1:**
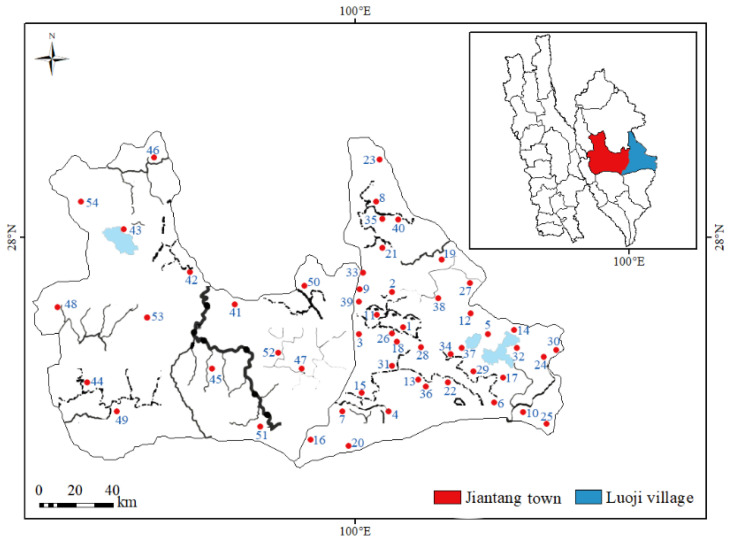
Distribution of 54 sampling sits in Luoji (n = 40) and Jiantang (n = 14), Yunnan Province, Southwest China.

Paired soil (0–10 cm) and *T*. *matsutake* (n = 54) samples were collected from two geographic villages in Diqing State, Yunnan Province, China, during mushroom harvest season, including Luoji (n = 40) and Jiantang (n = 14) (Figure 1). Each soil or *T*. *matsutake* sample was made up of five well-mixed subsamples. Soils were air-dried, ground, well mixed and passed through a 100-mesh (0.15 mm) nylon sieve. Fresh *T*. *matsutake* fruiting bodies were rinsed with deionized (DI) water to remove surface-adsorbed soil and elements, then separated into caps and stipes and lyophilized at −80 °C to constant weights (FreezZone 12, LabConco, Kansas City, MO, USA). Freeze-dried mushrooms were ground under liquid nitrogen to obtain homogeneous powders and stored at −20 °C before further analyses.

### 2.2. Chemical Analysis

The soil pH was determined by mixing soil with 0.01 M CaCl_2_ solution at 1:5 (*m*/*v*), shaking at 180 rpm and 25 °C for 1 h, then analyzing the supernatant with a pH meter (Mettler–Toldo, Columbus, OH, USA) [18]. The soil organic matter (OM) content was determined gravimetrically after combustion at 550 °C for 16 h in a furnace horn (Select–Horn, SELECTA, Cham, Switzerland) [19].

Metal concentrations in soils and *T*. *matsutake* were analyzed with X-ray fluorescence (XRF, E-max500, Z–spec, Inc., East Greenbush, NY, USA) under normal detection mode. The radio frequency power was 1050 W and the measuring time was 600 s [20]. Standard reference materials including the mushroom *Lentinus edodes* (GBW10197) and soil (GSS1) were used for concentration assays for quality assurance and quality control. The Fe, Mn, Zn and Cu concentrations obtained via XRF for GBW10197 were 141 ± 0.61, 26.2 ± 0.16, 53 ± 0.17 and 6.23 ± 0.05 mg kg^−1^ (mean ± SD, n = 3), which were in good agreement with the certified values at 152 ± 21, 25 ± 0.8, 51 ± 3.8 and 5.73 ± 0.18 mg kg^−1^. The Mn, Zn and Cu concentrations obtained via XRF for GSS1 were 1644 ± 5.86, 630 ± 0.7 and 20.9 ± 0.46 mg kg^−1^, which were in good agreement with the certified values at 1760 ± 63, 680 ± 25 and 21 ± 2 mg kg^−1^. The average recoveries were 92.6–109%. All analyses were performed in triplicate.

### 2.3. Bioaccumulation and Translocation Analysis

To evaluate the metals accumulation from soil to the *T*. *matsutake* cap and stipe, the bioaccumulation factor (BAF) was calculated via Equation (1) [21]:

(1) $$BAF=\frac{C_{mushroom}}{C_{soil}}$$

where C_mushroom_ is the concentration of the individual metal in the *T*. *matsutake* cap or stipe (mg kg^−1^) and C_soil_ is the corresponding metal concentration in the soil (mg kg^−1^). BAF > 1 indicates that the organism is an accumulator towards the given element.

To estimate the metals transfer and distribution from the *T*. *matsutake* stipe to cap, the translocation factor (TF) was calculated via Equation (2) [22]:

(2) $$TF=\frac{C_{c}}{C_{s}}$$

where C_C_ is the concentration of the individual metal in the cap and C_S_ is the concentration in the stipe.

### 2.4. Health Risk Analysis

To assess the potential health risk of human exposure to metals-contaminated *T*. *matsutake*, the health risk index (HRI) was analyzed via Equation (3) [23]:

(3) $$HRI=\frac{DI}{{R_{f}D}_{i}}$$

where DI is the daily intake of metal per kg of human body weight (bw) via *T. matsutake* consumption (µg kg^−1^ bw d^−1^; Equation (4)). R_f_D_i_ is the reference dose of oral intake of the metal (i) (µg kg^−1^ bw d^−1^) proposed by the Joint FAO/WHO Expert Committee on Food Additives (JECEFA) and the US Environmental Protection Agency (USEPA) [24,25]. The R_f_D values established for Fe (JECEFA), Mn, Zn and Cu (USEPA) were 300, 140, 300 and 40 µg kg^−1^ bw d^−1^ (Table 1) [26].

The daily intake (DI; µg kg^−1^ bw d^−1^) of metals was calculated via Equation (4) [6]:

(4) $$DI=\frac{SM\times MCM}{BW}$$

where SM is a daily serving amount (0.03 kg dried *T. matsutake*), MCM is the metal concentration in the mushrooms (mg kg^−1^ dw) and BW is the average human body weight (70 kg for adults) [27]. The provisional tolerable maximum daily intake (PTMDI) values for Cu and Zn were 300–1000 and 5000 µg kg^−^^1^ bw d^−^^1^ (JECEFA) (Table 1).

### 2.5. Statistical Analysis

Results are presented as the mean of triplicate analyses and standard deviation. Statistical differences and variance was evaluated by one-way ANOVA and Duncan’s multiple range tests at *p* < 0.05 (SPSS 20.0, SPSS Corporation, New York, NY, USA). Pearson correlation analysis was established by SPSS 25.0 at *p* < 0.05 or *p* < 0.01. The figures were drawn using Origin 2022 (Origin Lab Corporation, Northampton, MA, USA).

## 3. Results and Discussion

### 3.1. Soil pH, OM and Metals Concentration

Among soil characteristics, the pH, OM content and metal total concentration are critical in mediating metal uptake and accumulation in mushrooms [28]. The soil pH values were 3.95–6.56, indicating that *T*. *matsutake* prefers to grow in an acidic environment (Table 1). Normally, Southwest China is prevalent with karst soils with pH values at 6.07–8.53 [29]. Given that mushrooms usually grow in the forest, the acidic to weak acidic soils may be attributed to litter decomposition, which produces organic acids to contribute protons [30]. Soil acidification renders metals mobilized and released into the soil solution, which are readily taken up by mushrooms [31]. This was in consistent with the finding that forest soils growing wild mushrooms are acidic in Poland, with pH values low at 3.35 in pine understory soils [32].

Soil OM is the organic fraction originated from plant and animal decomposition and microbial activities [33]. OM is a strong metal sorbent in organic forest soils for the rich carboxyl and hydroxyl groups, which can complex with metal cations and affect their mobility and bioavailability [34,35]. In this study, soil OM showed strong heterogeneity ranging from 1.29% to 44.5% (Table 1), which was higher than the reported values in agricultural soils of Yunnan (1.67–9.78%) and South Africa (1.5–13.7%) [31]. Generally, metal bioavailability decreases with increasing OM due to the strong adsorption, complexation and chelation [36,37]. However, evidence suggests that metal bioavailability can be increased with increasing OM, attributed to the fact that OM chelates metal to form soluble organo-metal complexes [38]. This was supported by the finding that soil Cu and Zn bioavailability was significantly (*p* < 0.05) positively correlated with OM content (R = 0.73 and R = 0.86) [39].

**Figure 2:**
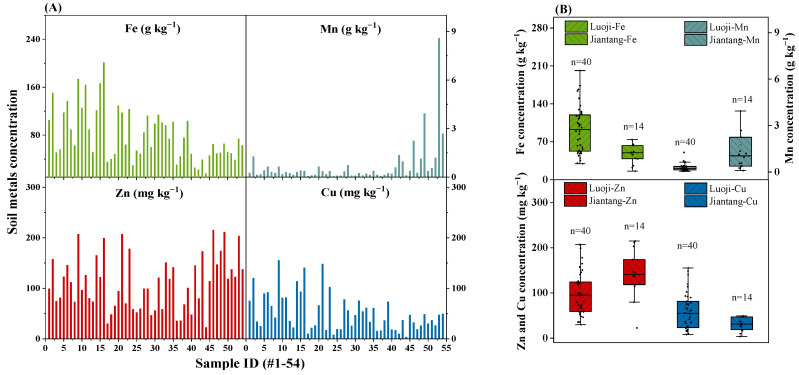
Metals (Fe, Mn, Zn, Cu) concentration in soils (**A**) and variations between the regions of Luoji (n = 40) and Jiantang (n = 14) (**B**). The bottom and top of the box represent the 25th and 75th percentiles and the error bars represent the minimum and maximum values within the normal range. The solid lines inside the box represent the median value.

The metals concentrations in *T*. *matsutake* growing soils showed a strong heterogeneity, with Fe and Mn (16–201 and 0.046–8.58 g kg^−1^) being much higher than Zn and Cu (22.6–215 and 3.7–155 mg kg^−1^) (Figure 2). The soil metal concentrations in this study were higher than soils growing wild mushrooms (*Macrolepiota procera*, *Imleria badia*, *Leccinum scabrum* and *Boletus edulis*): Fe (0.12–5.36 g kg^−1^), Mn (0.014–0.12 g kg^−1^), Zn (3.75–31 mg kg^−1^) and Cu (0.24–21 mg kg^−1^) [32]. This may be due to the intensive activities of energy and raw material industries like coal, electricity, petrochemical and nonferrous metals in Yunnan Province [40]. In forested areas, soil metals often originate from parent material and atmospheric deposition (busy roads and emitters). The high concentration of metals in soils may be transferred to *T*. *matsutake*, so the metals concentrations and bioaccumulation in *T*. *matsutake* were examined.

### 3.2. Metals Concentration and Distribution in T. matsutake

#### 3.2.1. High Metals Concentration in *T. matsutake*

**Figure 3:**
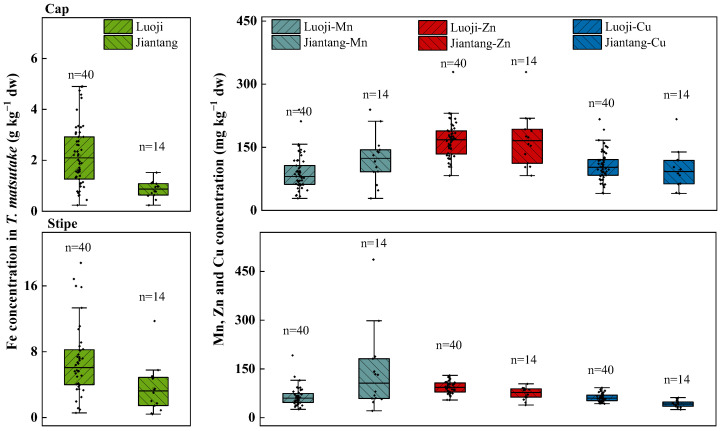
Metals (Fe, Mn, Zn, Cu) concentrations in *T*. *matsutake* cap and stipe and comparisons between the regions of Luoji (n = 40) and Jiantang (n = 14). The bottom and top of the box represent the 25th and 75th percentiles and the error bars represent the minimum and maximum values within the normal range. The solid lines inside the box represent the median value.

Given the high concentration of Fe in soils, the Fe concentration in *T*. *matsutake* was the highest among the four metals (0.24–18.8 g kg^−1^ vs. 21.1–487 mg kg^−1^) especially in Luoji village (Figure 2B). However, despite the much higher concentration of Mn than Zn and Cu in soils (0.046–8.58 g kg^−1^ vs. 22.6–215 and 3.7–155 mg kg^−1^) (Figure 2A), their concentrations in *T*. *matsutake* were comparable (21.1–487 vs. 38.7–329 and 24.9–217 mg kg^−1^) (Figure 3). This indicated that *T*. *matsutake* prefers to accumulate Zn and Cu compared to Mn.

Generally, Fe is an essential mineral element for mushrooms [13]. The Fe concentration in *T*. *matsutake* (0.24–18.8 g kg^−1^; Figure 3) was higher than that in *T*. *matsutake* collected from Sichuan Province, China (0.01–0.08 g kg^−1^) [41]. In addition, it was much higher than a wide range of wild mushroom species (*Lactarius deliciosus*, *Clitocybe houghtonii*, *T. argyraceum* and *B. chrysenteron*) at 0.16–0.43 g kg^−1^ [42] and a large sample size (*Amanita rubescens*, *Suillus granulatus*, *Bovista plumbea* and *Lycoperdon perlatum*) at 0.02–0.17 g kg^−1^ (n = 102) from unpolluted areas with a soil Fe concentration at 14.4–27 g kg^−1^ [43]. This suggested that soil Fe is an important source for its accumulation in wild mushrooms. This was supported by the finding in Jilin Province, China, showing that the high As concentration in *T. matsutake* from Changbai Mountain was due to the high As content in black soils [44]. Still, compared within Yunnan Province, the *T*. *matsutake* Fe concentration in the present studied areas Luoji (0.57–18.8 g kg^−1^) and Jiantang (0.24–11.7 g kg^−1^) was higher than that from Lijiang, Nanhua, Zhongshan and Deqin (0.046–0.42 g kg^−1^) with lower soil Fe contents at 0.29–3.07 g kg^−1^ [6].

Though the soil Mn concentration was orders of magnitude higher than Zn and Cu, their concentrations in *T*. *matsutake* were comparable. Specifically, the Mn concentration in *T*. *matsutake* was 21.1–487 mg kg^−1^ (Figure 3), which was higher than that in *T*. *matsutake* (1.54–29.4 mg kg^−1^) from Lijiang, Nanhua, Zhongshan and Deqin, Yunnan Province [6], and other species including *Coprinus comatus*, *Voluariella volvacea* and *Pleurotus nebrodensis* at 13.5–113 mg kg^−1^ [5]. Similarly, the Zn concentration in the present study (38.7–329 mg kg^−1^) was higher than that in *T*. *matsutake* (8.71–46.9 mg kg^−1^) [6] and *M*. *procera* (22–240 mg kg^−1^) [45] but was comparable with that in *Agaricus bisporus*, *B*. *edulis* and *T. columbetta* (30–310 mg kg^−1^) [46].

Similarly to Mn and Zn, the Cu concentration (24.9–217 mg kg^−1^) was much higher than the reported values in *T*. *matsutake* from four regions in Yunnan Province at 1.53–12.6 mg kg^−1^, which may be attributed to the different concentrations in soils at 3.7–155 (Figure 2) vs. 26.5–51.9 mg kg^−1^ [6]. In addition, it was higher than the Cu concentrations (7.3–123 mg kg^−1^) in 20 wild mushroom species grown in the “green lung region” of Poland without urbanization or industry [47]. However, it was also higher than in wild mushrooms (*L*. *scabrum*, *B. reticulatus* and *L. griseum*) (17.1–162 mg kg^−1^) collected in a highly contaminated area in Eastern Slovakia [48].

As such, the data indicated that the metals concentration in the studied *T*. *matsutake* was much higher than other mushroom species from other regions and the same species in the same province. Therefore, the underlying influencing factors and potential health risks to humans should be studied.

#### 3.2.2. Metals Distribution in Cap and Stipe

Fe was mainly stored in the stipe (69.1%), while Mn, Zn and Cu were transferred to the cap (54.1%, 65.3% and 64.1%) (Figure 3), indicating that *T*. *matsutake* was more efficient in transferring Mn, Zn and Cu than Fe. Specifically, the Fe contents in the cap and stipe were 0.24–4.9 and 0.41–18.8 g kg^−1^, with the average value at 2.2 and 6.02 g kg^−1^. A greater Fe content in the stipe (0.26 g kg^−1^) than in the cap (0.08 g kg^−1^) was also found in *M*. *procera* (n = 15) [45].

In contrast, the Mn, Zn and Cu content in the cap (21.1–487, 82.8–329 and 40.5–218 mg kg^−1^) was much higher than in the stipe (28.7–239, 38.7–130 and 24.9–92.1 mg kg^−1^). A similar result was found in *Amanita muscaria*, showing a greater Zn content in the cap (150–250 mg kg^−1^) than in the stipe (110–240 mg kg^−1^) [49]. Similarly, a greater cap than stipe content of Cu was found in wild mushrooms (*B*. *edulis*, *B*. *reticulatus*, *L*. *scabrum* and *L*. *griseum*) at 35.2–162 vs. 17.1–72.4 mg kg^−1^ [48]. This suggested that a greater stipe-to-cap transfer of Zn and Cu may be common in wild mushrooms including *T*. *matsutake*.

### 3.3. Metals Bioaccumulation and Transfer in T. matsutake

**Figure 4:**
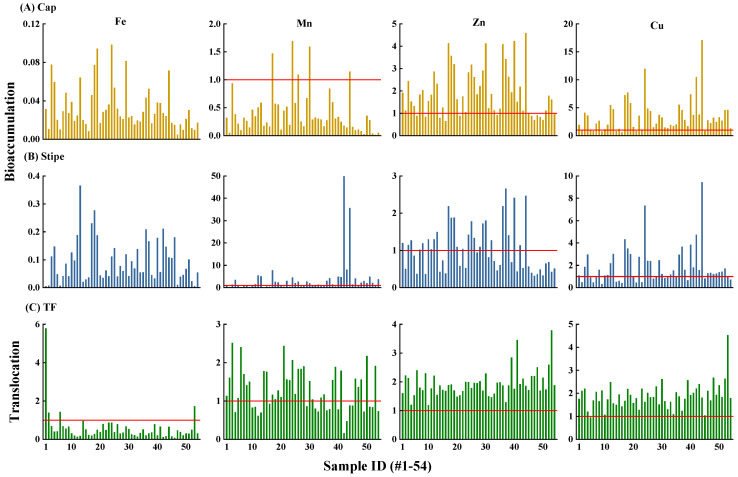
Bioaccumulation factor (BAF) of Fe, Mn, Zn and Cu in cap (**A**) and stipe (**B**) and the translocation factor (TF) (**C**) in *T*. *matsutake* (n = 54). BAF > 1 and TF > 1 (red line) indicates that *T*. *matsutake* possesses accumulating or stipe-to-cap translocating ability towards the given element, respectively.

Though *T*. *matsutake* accumulated high concentrations of Fe (Figure 3), the bioaccumulation factor (BAF = 0.005–0.1) suggested that it is not a hyperaccumulator (BAF < 1) towards Fe (Figure 4A,B). Similarly, *T*. *matsutake* was also not a Mn hyperaccumulator with 91.7% of samples showing BAF < 1. Instead, *T*. *matsutake* can hyperaccumulate Zn and Cu, with 63% and 77.8% of samples showing BAF > 1 and reaching 4.59 and 17.1. The ability of wild mushrooms to accumulate metallic elements is related to the network of hyphae located in the upper soil horizon [50,51]. Hyphae consisting of elongated tubular cells enveloped by a chitin wall are widely spread over the bioavailable areas to accumulate metal ions [52]. In addition, this process is influenced by environmental factors (soil metal concentration, pH and OM) and intrinsic properties (size and mycelial age) [53].

Consistent with the metals distribution (Figure 3), the average stipe-to-cap translocation factors (TFs) of Zn and Cu were higher than those of Fe and Mn (1.94 and 1.89 vs. 0.58 and 1.28) (Figure 4C). Specifically, the percentage of TF > 1 was 100% and 98% for Zn and Cu, while that for Fe and Mn was 7.4% and 63%. The greater translocation of Zn and Cu was consistent with but higher than the reported values in *M*. *procera* (TF = 1.22–2.07 and 0.55–1.76) [54]. This may be due to the different nature and concentration of proteins between the cap and the stipe, which was evidenced by the various carpophore structures showing a more complex electrophoretic spectrum in the cap than the stipe [54,55].

### 3.4. Potential Risk to Human Health

Mineral metals are essential components for human health, which, however, exert toxic effects when exceeding the amount required for physiological functions [1]. To evaluate the potential health risk associated with *T*. *matsutake* consumption, the daily intake (DI) of metals and the health risk index (HRI) were analyzed.

#### 3.4.1. Metals Daily Intake Estimate

The daily intake (DI) of metal was calculated and compared with certificated values proposed by JECEFA and USEPA. The reference dose (R_f_D) values established for Fe (JECEFA), Mn, Zn and Cu (USEPA) were 300, 140, 300 and 40 µg kg^−1^ bw d^−^^1^ (Table 1). The provisional tolerable maximum daily intake (PTMDI) values for Zn and Cu were 300–1000 and 5000 µg kg^−1^ bw d^−^^1^ (JECEFA).

Among the four metals, Fe showed the highest DI values (102–8058 µg kg^−1^ bw d^−1^), especially for the Luoji region at 244–8058 µg kg^−1^ bw d^−1^ (Table 1). Typically, 93.5% of DI values for Fe in *T*. *matsutake* exceeded the R_f_D limit (300 µg kg^−^^1^ bw d^−^^1^). In contrast, the DI values for Mn (99.1%) and Zn (100%) were generally within the R_f_D limits. In terms of Cu, 30.6% of DI values exceeded the R_f_D limit established by USEPA (40 µg kg^−^^1^ bw d^−^^1^), but all were within the PTMDI limit established by JECEFA (5000 µg kg^−^^1^ bw d^−^^1^). This suggested that the daily intake of Fe via *T*. *matsutake* consumption may cause a risk to human health.

The DI values in the present study were generally higher than the reported values in the wild mushrooms *Amanitaceae*, *Lactarius* and *Russulaceae* but lower than for *Agaricaceae* from different regions including Spain and Morocco [56,57]. For example, the average DI value for Mn in this study was 5-fold that of 13 wild mushroom species from Belgrad forest at 41.2 vs. 8.16 µg kg^−^^1^ bw d^−^^1^ [58]. The difference can be attributed to regional soil geochemical characteristics and the physiology and genetic characteristics of individual mushroom species [1].

Further, thermal cooking processes were reported to increase the metals concentration in mushrooms. For example, frying increased the Fe and Mn content in *A*. *bisporus* (n = 540) from 66.2 to 69.5 and from 5.77 to 7.0 mg kg^−1^ dw. Boiling and frying increased the Zn and Cu content from 126 to 153–156 and from 56.4 to 59.4–60 mg kg^−1^ dw [59]. This suggested that high-temperature processing may increase the risk of metals via wild mushroom ingestion, so the detailed effects and toxic mechanisms deserve further investigation for risk control during food preparation.

#### 3.4.2. Health Risk Assessment

**Figure 5:**
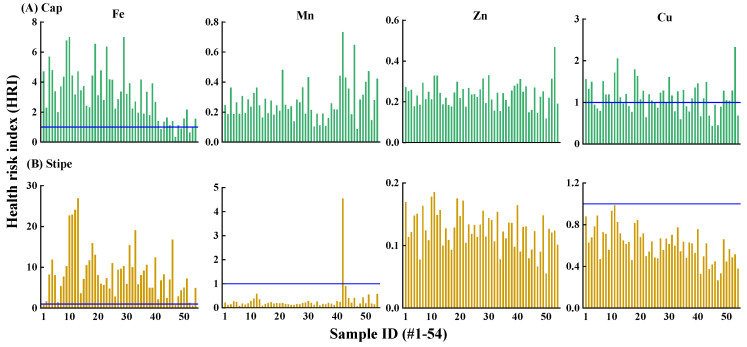
Health risk index (HRI) of Fe, Mn, Zn and Cu via ingestion of *T*. *matsutake* cap (**A**) and stipe (**B**) (n = 54). HRI > 1 (blue line) indicates there is a potential health risk of the element via consumption of the *T*. *matsutake* cap or stipe.

A health risk index (HRI) > 1 for a given metal indicates there is potential risk to human health [6,60]. Consistent with the high concentration and high DI value in *T*. *matsutake*, Fe showed the highest risk with 93.5% of HRI > 1 (Figure 5). Especially since Fe was mainly stored in the stipe (Figure 3), stipe Fe showed a greater risk than in the cap with HRI values of 0.59–26.9 vs. 0.34–7.0 (Figure 5). The Fe HRI value in this study was lower than that in wild mushrooms (*Amanita mellea*, *Hygrophorus pudorinus*, *Polyporus squamosus* and *Russula vinosa*) from Turkey, Spain, and Morocco at 21.4–97 [60]. HRI values suggested that Fe in wild mushrooms from specific geographical locations may exert a health risk to humans.

Compared to Fe, Mn (99.1%) and Zn (100%) in *T*. *matsutake* showed no potential health risk (Figure 5). Cu is an essential element occurring in enzymes that is important in the immune and nervous systems [61]. Nonetheless, it may still pose a risk to human health at elevated levels of exposure. The result indicated that 61.1% of Cu in the cap showed a risk with an HRI value at 1.01–2.33. The higher risk of Cu than Zn was consistent with *M*. *procera* from Southern Spain and Northern Morocco, showing that the HRI of Cu was >1, while that of Zn was <1 [54]. As such, Fe in *T*. *matsutake* showed the greatest risk, followed by the cap Cu, while Mn and Zn were considered risk-free.

### 3.5. Correlation between Soil Properties and T. matsutake Metals Accumulation

**Figure 6:**
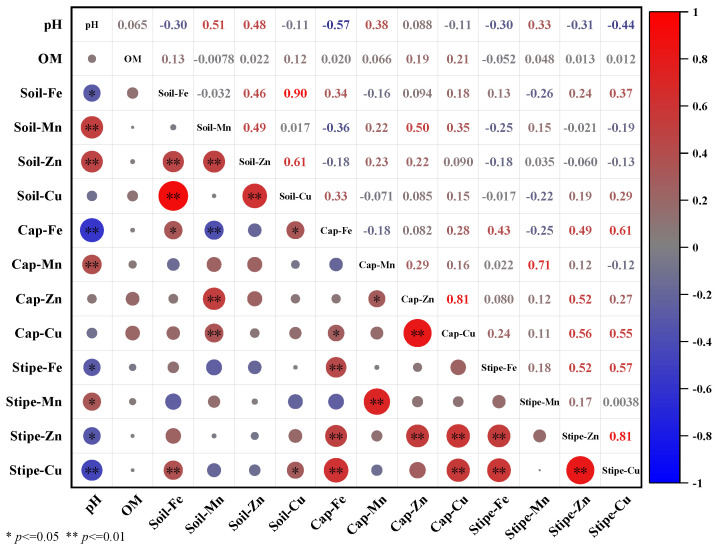
Correlations of metals (Fe, Mn, Zn and Cu) concentration in *T*. *matsutake* cap and stipe with soil pH, organic matter content (OM) and metals concentration with significance at *p* < 0.05 (*) or *p* < 0.01 (**).

The metals bioavailability in soils and accumulation in *T*. *matsutake* depend on the metal total concentration, soil pH and OM; therefore, the correlations between soil and *T*. *matsutake* were analyzed (Figure 6).

The result showed that the soil metals total concentration and pH showed significant effects on metals accumulation in *T*. *matsutake*, especially on the cap Fe and stipe Cu. Specifically, the cap Fe was significantly positively affected by the soil Fe content (R = 0.34, *p* < 0.05), while it was negatively affected by the soil pH (R = −0.57, *p* < 0.01). Similar to the cap Fe, the stipe Cu was significantly positively correlated with the soil Cu content (R = 0.29, *p* < 0.05), while it was negatively correlated with the soil pH (R = −0.44, *p* < 0.01). In addition, the soil Cu content showed a greater effect on its accumulation in the stipe than in the cap (R = 0.29 vs. −0.15). This was consistent with Su et al., finding that the correlation of the soil Cu concentration with that in *B*. *edulis* stipe and cap was R = 0.65 and −0.13 [62]. This again suggested that soil is an important source for Fe and Cu accumulation in the *T*. *matsutake* cap and stipe, respectively, and acidic soils (pH = 3.95–6.56; Table 1) further increase their mobility and accumulation [62].

Contrary to Fe and Cu, both the cap (R = 0.38, *p* < 0.01) and stipe Mn (R = 0.33, *p* < 0.05) were significantly positively correlated with soil pH. Therefore, acidic conditions may decrease Mn accumulation in *T*. *matsutake*. This was supported by the very high Mn content in soils (0.046–8.58 g kg^−1^; Figure 2A) while low accumulation in *T*. *matsutake* (21.1–487 mg kg^−^^1^; Figure 3). In addition, the cap and stipe Mn were positively correlated with the soil Mn content (R = 0.22 and 0.15), which were weaker than that in *B*. *badius* (R = 0.34 and 0.43) [63]. In terms of Zn, the cap was positively correlated with the soil Zn concentration (R = 0.09) and pH (R = 0.09), while the stipe showed a significant negative correlation with the soil pH (R = −0.31, *p* < 0.05). This indicated that the soil Mn and Zn concentrations exert relatively low effects on their accumulation in *T*. *matsutake*.

## 4. Conclusions

This study investigated four metals (Fe, Mn, Zn and Cu) concentrations, translocation and accumulation from soil to *T*. *matsutake* and evaluated the potential health risk of the metals via *T*. *matsutake* ingestion. The results showed that the metals concentrations in *T*. *matsutake* growing soils were strongly heterogenous. The Fe and Mn (16–201 and 0.046–8.58 g kg^−1^) concentrations were much higher than those of Zn and Cu (22.6–215 and 3.7–155 mg kg^−1^). The highest Fe concentration in the *T*. *matsutake* cap (0.24–18.8 g kg^−1^) and the significant positive correlation with the soil Fe content (R = 0.34, *p* < 0.05) suggested that soil Fe is an important source for its accumulation in *T*. *matsutake*. In contrast to Fe, the high concentration of Mn in soils does not necessarily lead to high accumulation in *T*. *matsutake*, with the BAF at 0.006–1.69. In addition, *T*. *matsutake* showed accumulation and transfer ability towards Zn and Cu, where BAF and TF were 0.32–17.1 and 0.96–4.53. Correspondingly, Fe showed the highest health risk with 92.6–94.4% of samples showing an HRI > 1. In addition to the soil Fe concentration, the high Fe accumulation in *T*. *matsutake* and the high potential risk were related to the low soil pH (3.95–6.56), which were significantly negatively correlated (R = −0.57, *p* < 0.01).

## Figures and Tables

**Table 1:** Soil pH and organic matter content and daily intake (DI) of Fe, Mn, Zn and Cu via ingestion of *T*. *matsutake* cap and stipe from Yunnan Province, Southwest China.

Sample ID	Soil pH	Soil Organic Matter Content (%)	DI (µg kg^−1^ bw d^−1^)							
			Fe		Mn		Zn		Cu	
			Cap	Stipe	Cap	Stipe	Cap	Stipe	Cap	Stipe
1	4.57	6.96	1413	244	34.7	30.5	81.6	50.9	62.1	35.2
2	5.33	11.9	689	493	26.0	16.2	75.6	34.1	53.1	25.2
3	4.32	8.79	1708	2464	50.9	20.3	77.8	36.5	59.9	27.1
4	4.39	10.6	1437	3564	26.4	37.2	53.2	44.3	37.8	31.4
5	4.64	13.7	1013	2412	37.0	34.4	69.3	45.2	34.0	35.5
6	4.58	9.63	596	416	26.5	11.0	55.8	23.3	31.8	18.8
7	4.32	9.58	1115	1613	43.0	25.3	88.0	49.0	60.3	29.1
8	4.99	18	1303	2301	27.0	19.2	63.7	37.3	47.4	28.5
9	5.02	27.8	2033	3077	39.9	26.6	74.8	32.5	47.6	22.4
10	4.19	3.86	2097	6799	33.0	39.8	63.5	53.4	39.8	37.4
11	5.06	11.4	1329	6857	45.8	54.0	98.6	55.6	68.5	39.5
12	4.06	7.71	948	7216	50.8	82.2	98.7	44.6	82.2	33.0
13	4.32	7.87	1416	8058	34.3	49.2	73.0	47.1	44.8	28.7
14	4.74	10.6	1033	1073	22.8	12.9	56.2	30.0	39.1	26.0
15	4.23	1.29	1119	2132	40.4	22.9	65.9	38.2	48.1	24.6
16	4.84	5.07	727	3145	26.8	29.0	55.2	32.7	36.3	25.3
17	4.68	6.84	697	3495	38.6	33.1	52.9	28.0	30.8	18.4
18	4.83	15.1	1330	4760	25.5	24.4	73.7	38.7	71.6	32.7
19	4.8	6.23	1964	3912	34.3	26.9	89.4	52.6	65.4	33.8
20	4.31	17.3	935	2419	29.2	26.4	65.5	44.1	43.0	27.2
21	4.81	3.77	1434	1784	67.4	27.7	79.0	51.5	51.2	28.7
22	4.13	6.21	836	1695	34.8	22.3	52.7	31.4	25.6	19.9
23	4.52	11.1	1907	2185	30.6	19.9	80.4	40.1	47.7	21.7
24	4.26	2.78	1253	1427	33.3	16.1	70.7	35.5	41.7	25.6
25	4.12	7.55	1246	3285	19.4	16.4	71.1	39.9	39.3	19.4
26	4.1	3.85	667	840	39.7	21.6	67.0	34.1	34.9	19.1
27	4.7	6.2	860	2811	37.1	20.1	78.5	40.1	49.2	26.8
28	4.48	6.68	1008	2877	51.1	26.8	94.2	46.7	51.3	22.3
29	4.44	3.09	2099	3081	26.5	30.7	58.1	34.3	40.5	26.7
30	4.19	44.5	962	1764	60.7	40.0	99.1	43.2	64.4	24.6
31	4.57	8.4	1178	4609	29.9	28.7	63.3	42.2	46.6	28.2
32	4.2	8.61	669	2996	14.5	18.1	46.9	32.0	31.4	23.9
33	4.55	10.3	810	5716	26.4	36.5	73.3	46.1	50.5	31.0
34	4.74	8.76	584	1733	15.6	14.3	46.2	23.4	23.8	21.8
35	3.95	12.2	1250	2414	26.7	22.8	72.8	36.7	52.0	25.4
36	4.89	10.6	566	2739	15.2	20.0	62.5	33.4	36.1	19.3
37	4.66	8.77	1005	3172	22.5	28.7	52.9	41.0	31.2	25.1
38	4.68	5.59	539	1485	36.0	23.3	76.5	40.8	43.8	24.8
39	4.55	7.71	1175	1479	30.5	16.2	83.5	29.4	54.1	21.1
40	4.68	10.6	800	3722	30.4	39.0	86.6	49.3	58.3	30.3
41	6.37	5.53	417	627	61.8	34.5	93.6	27.1	26.6	13.1
42	5.52	10.3	261	2027	103	637	74.7	38.9	44.0	19.8
43	5.94	13.4	408	2473	60.1	128	82.7	39.2	59.6	24.8
44	5.08	7.12	490	744	49.9	56.5	44.5	23.9	27.1	15.0
45	6.29	4.48	335	2092	26.0	29.4	48.0	28.0	17.4	16.7
46	6.11	8.74	418	5031	90.8	57.3	81.1	36.9	37.8	17.9
47	4.79	7.49	102	217	12.3	9.0	43.9	20.0	18.0	10.7
48	5.96	10.6	334	861	39.6	25.3	67.5	27.0	35.8	13.3
49	6.3	6.51	273	1293	44.1	61.0	75.3	44.5	51.1	26.4
50	5.96	12.9	472	1502	56.2	25.8	35.5	16.6	42.2	17.9
51	5.38	3.94	650	2156	66.1	77.7	66.0	38.1	41.6	22.6
52	6.25	21.3	192	385	20.5	24.5	93.7	36.2	51.3	19.5
53	5.79	7.12	306	177	39.3	20.6	141	37.1	93.2	20.6
54	6.56	15.3	465	1476	59.1	80.6	57.2	30.4	27.3	15.2
R_f_D ^a^ (µg kg^−^^1^ bw d^−^^1^)			300 (JECEFA) ^c^		140 (USEPA) ^d^		300 ^d^		40 ^d^	
PTMDI ^b^ (µg kg^−^^1^ bw d^−^^1^)			–		–		300–1000 ^c^		5000 ^c^	
Percentage of samples exceeding R_f_D (%)			92.6%	94.4%	0	1.85%	0	0	61.1%	0
Percentage of samples exceeding PTMDI (%)			–		–		0	0	0	0

^a^ R_f_D: Reference dose. ^b^ PTMDI: Provisional maximum tolerable daily intake. ^c^ JECEFA: The Joint FAO/WHO Expert Committee on Food Additives. ^d^ USEPA: US Environmental Protection Agency.

## Data Availability

The original contributions presented in the study are included in the article, further inquiries can be directed to the corresponding author.
